# Oral manifestation of Langerhans cell histiocytosis: a case report

**DOI:** 10.1186/s12903-018-0568-5

**Published:** 2018-06-08

**Authors:** Julia Luz, Daniel Zweifel, Martin Hüllner, Marco Bühler, Martin Rücker, Bernd Stadlinger

**Affiliations:** 1 Clinic of Cranio-Maxillofacial and Oral Surgery, University of Zurich, University Hospital Zurich, Plattenstrasse 11, CH-8032 Zurich, Switzerland; 2 Department of Nuclear Medicine, University Hospital Zurich, Zurich, Switzerland; 3 Institute of Pathology and Molecular Pathology, University Hospital Zurich, Zurich, Switzerland

**Keywords:** Histiocytosis, Langerhans-cell, Eosinophilic granuloma, Osteonecrosis

## Abstract

**Background:**

Bone necrosis of the jaw is a serious condition with a broad differential diagnosis of pathologies such as cutaneous histiocytosis, bone metastases or malignant tumours. In addition to the most common cause, medication related osteonecrosis of the jaw (MRONJ), one must consider a number of other causes, such as histiocytosis.

Langerhans cell histiocytosis (LCH) is a histiocytic disorder with a large spectrum of clinical manifestations and with possible involvement of a variety of organs. This case shows the importance of an early detection of this rare disease in order to prevent further spreading. Even if an initial diagnosis in the oral cavity is rare, dentists should be aware of this disease.

**Case presentation:**

The presented case describes a patient who was referred for evaluation and treatment due to exposed bone and extensive osteolysis in the region of the upper and lower jaw. After biopsy and diagnosis of LCH, the patient was treated with systemic therapy, achieved remission and is disease free after a 2 year of follow up.

**Conclusions:**

This case report illustrates that when dealing with unclear osteolytic changes of the jawbone, Langerhans cell histiocytosis must be taken into consideration in the differential diagnosis and biopsy must be performed in case of suspicion.

## Background

Langerhans cell histiocytosis (LCH), formerly known under a variety of different names such as histiocytosis X, eosinophilic granuloma, Hand-Schüller-Christian disease or Letterer-Siwe disease, is a rare disease of the family of histiocytosis characterized by the accumulation of histiocytic cells in various tissues. The discovery of Birbeck granules in the histiocytes of this disease and the expression of similar antigens has led to the name Langerhans cell histiocytosis. However newer findings show that LCH origins from dendritic myeloid progenitor cells rather than Langerhans cells of the skin [1]. LCH has a relatively higher incidence in children under the age of 15 years (5:1′000’000) but can also occur more rarely in adults (around 1:1′000’000). The etiology of the disease is still unknown, and there has been considerable debate whether LCH represents an inflammatory or a neoplastic disease. The discovery of recurrent mutations in the mitogen activated protein kinase (MAPK) pathway (i.e. *BRAF* and *MAP2K1* mutations) indicates that it is a neoplastic disease [2, 3]. Recently the Histiocyte Society has published a revised classification of histiocytoses in which LCH is sub classified according to site of manifestation and organ involvement: single system LCH, lung LCH and multi system LCH with or without risk organ involvement (risk organs: liver, spleen, bone marrow) [4]. Single system LCH affecting the skin or bone are the most frequent clinical manifestations, other less frequent sites of involvement include: pituitary gland, liver, spleen, bone marrow, lungs, lymph nodes and the central nervous system, although every organ can be affected. Lung LCH is considered separately in the classification, as it is very frequently associated with cigarette smoking, occurs predominantly in adults and is considered a form of interstitial lung disease [5]. A small, but not neglectable number of patients with multisystem, cranial or facial bone osteolytic lesions may develop diabetes insipidus, caused by lesions of the neurohypophysis or the supraoptic and paraventricular nuclei, sometimes representing the initial manifestation of the disease [6]. The clinical symptoms of LCH are usually seen as a secondary consequence of organ dysfunction. Skin lesions may present as papules or eczematous lesions, isolated or generealised [5]. Patients with bone LCH most often present localized pain and swelling of the affected area, sometimes with concurrent fever and the most commonly affected bones are the skull and the jawbone. In the oral region reported symptoms are gingivitis, periodontitis, tooth rotation or loss and malocclusion [7].

Possible differential diagnoses of LCH are other cutaneous histiocytoses such as xanthogranulomas, normolipemic granulomas, histiocytomas or haemophagocytic lymphohistiocytosis and should be considered [8]. In the present case the clinical presentation is difficult to be distinguished from medication related osteonecrosis of the jaw (MRONJ) or possible neoplastic lesions. Definite diagnosis will be achieved by the histopathological examination [9].

This case report informs the readership on methods of clinical and radiographic examination as well as treatment of this rare disease on the basis of this case. The importance of a thorough dental examination has also been underlined in a case of oral Multisystem LCH [10]. Although LCH is rare, it should be considered as a potential diagnosis.

## Case presentation

A 46-year-old male patient was examined in May 2015 due to tympanic effusion and right-sided hearing loss. The patient had a history of diabetes insipidus centralis of unknown aetiology, diagnosed in 2008. A ^18^F-fluoro-*D*-deoxyglucose (FDG) positron emission tomography /computed tomography (PET/CT) showed multiple FDG-avid lesions in the skull base, the temporal bone, the mandible and maxilla, but no lesions outside the head and neck area. Magnetic resonance imaging (MRI) of the same region showed a mass in the right-sided infratemporal fossa, erosions of the skull base and the alveolar ridge.

**Fig. 1:**
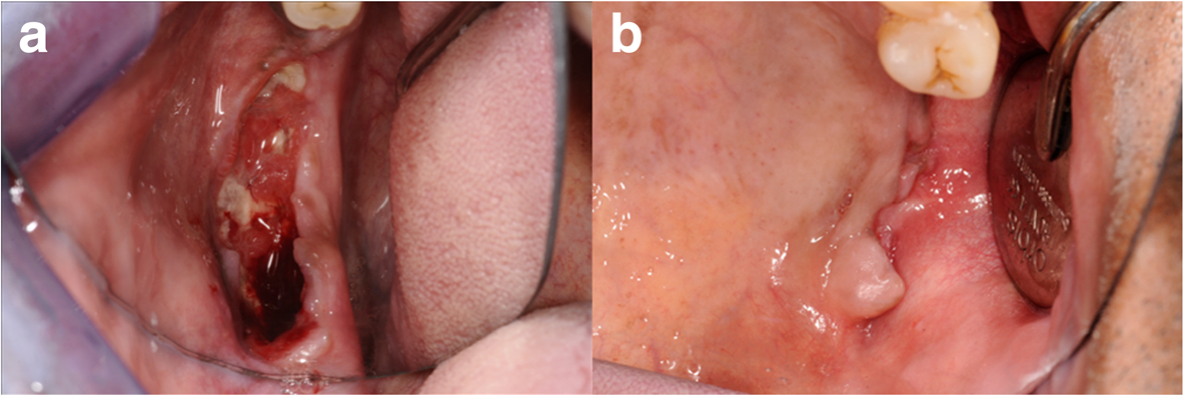
Clinical presentation of dehiscent gingiva in the mandible (**a**) and maxilla (**b**)

Due to the osteolysis of both the upper and lower jaw, the patient was transferred to our clinic. Here we conducted a cone beam computed tomography (CBCT) to determine the extent of the osteolysis. Clinically, an exposed alveolar ridge and ulcerative granulation tissue was present in region 45–47 and in the left upper jaw (Fig. 1a, b). Past medical history revealed that multiple teeth had been extracted by different dentists, and that the extraction sites showed prolonged or incomplete healing.

### Findings on magnetic resonance imaging (MRI)

Initially, the MRI showed an infiltrating mass in the central part of the right medial infratemporal fossa. There were osseous defects in other areas of the skull base and in the lower right alveolar ridge.

### Findings on positron emission tomography/computed tomography (PET/CT)

**Fig. 2:**
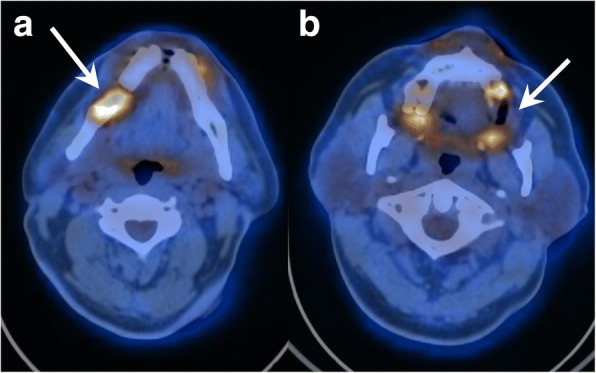
Baseline FDG-PET/CT scan shows lytic lesions in the right mandible (**a**, arrow) and left maxilla (**b**, arrow) with intense radiotracer uptake

**Fig. 3:**
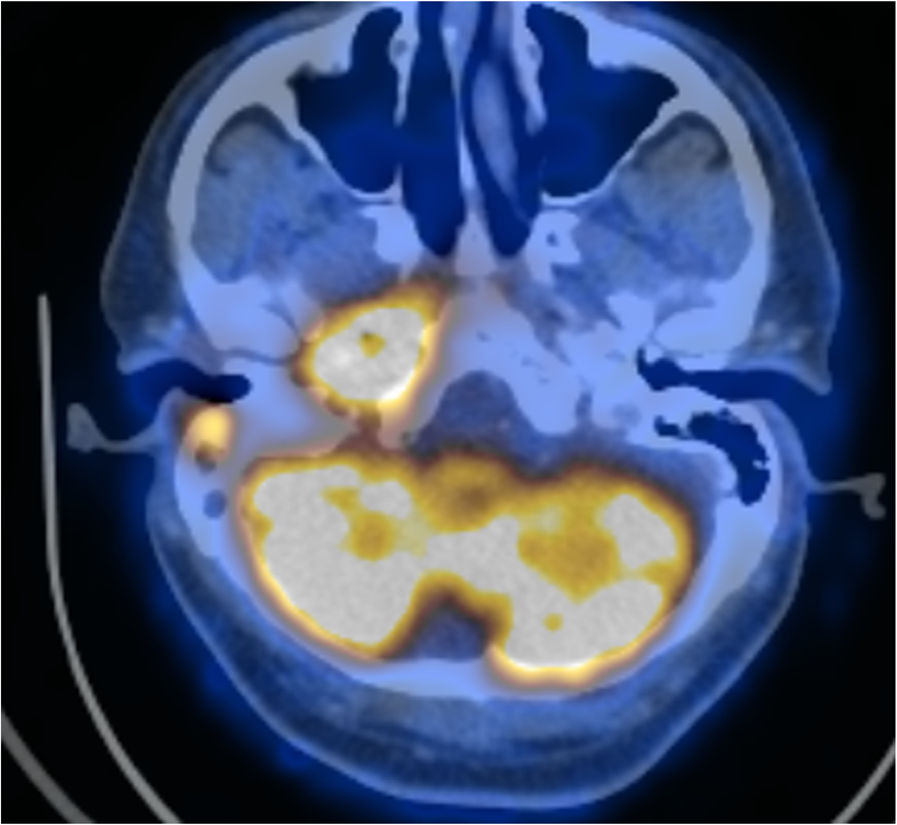
Coronal PET image in maximum intensity projection shows the FDG-avid lysis in the mandible on the right side and in the maxilla on the left side, as well as other FDG-avid lytic lesions in the right-sided mastoid process as well as in the right-sided skull base

The PET/CT of the skull, showed complete obliteration of the right middle ear and the mastoid cells as well as a soft tissue swelling in the external auditory canal of the right temporal bone. Further, a soft tissue lesion was seen in the right-sided skull base involving the temporal bone. FDG-PET/CT confirmed increased metabolic activity of the lesions in the right infratemporal fossa, in the tip of the mastoid and in the right horizontal mandibular ramus Fig. 2a, b). The localization of the other lesions is shown in Fig. 3. The biggest and most important lesion was situated in the right skull base with lysis of the petrous apex, the otic capsule, clivus, carotid canal, hypoglossal canal, jugular foramen and expansion in the retrostyloid space. No other clinical factors were present and there was no evidence of metabolically active local lymph nodes.

### Findings on cone beam computed tomography (CBCT)

**Fig. 4:**
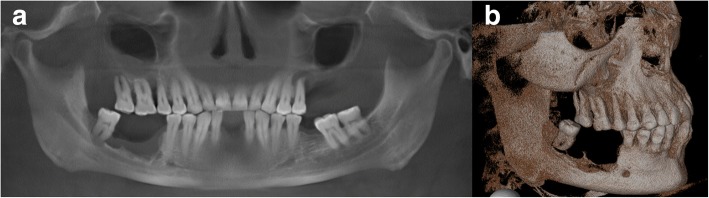
The baseline CBCT shows bone resorption in the right mandible and the left maxilla in a reconstructed panoramic view (**a**) and bone resorption in the right mandible in a 3D-reconstruction (**b**)

As the low-dose CT scan from PET/CT did not allow sufficient assessment of the mandibular bone structures, a CBCT was performed at our clinic (Accuitomo 170, Morita, Japan). The CBCT showed significant bone loss in the right mandible up to the level of the inferior alveolar nerve. In the left upper jaw, a complete resorption of the alveolar ridge was seen (Fig. 4a, b).

### Histopathological findings

**Fig. 5:**
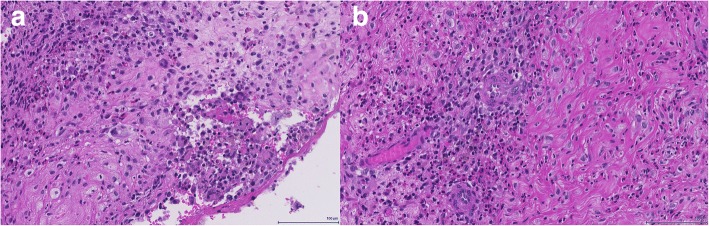
Photomicrographs show histiocytic infiltrates (**a**) with marked eosinophilia (**b**) (Hematoxylin and Eosin stain)

**Fig. 6:**
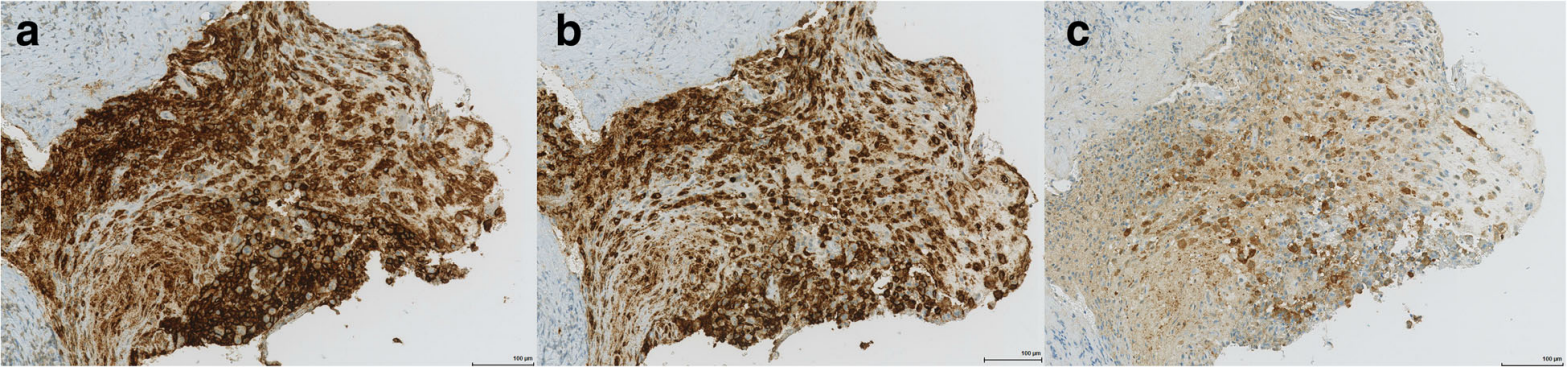
Immunohistochemistry shows the typical LCH phenotype CD1a (**a**), Langerin (**b**) and S-100 (**c**)

As the MRI showed a necrotic tumour in the region of the petrous apex, this area was selected for biopsy. Due to the delicate location of the bone lesion between the temporal bone and the middle ear, a preliminary computer assisted transnasal biopsy was carried out by the ENT department. The biopsy was taken from the pharyngeal recess in the right nasopharynx next to the petrous apex. Biopsy samples showed respiratory mucosa fragments with chronic and partially purulent inflammation with focal eosinophilia. No evidence of malignancy was seen, ancillary histochemical stainings showed no evidence of fungal infection. A second, more extensive excisional biopsy showed soft tissue with chronic inflammation and marked eosinophilia (Fig. 5). At high power magnification mononuclear histiocytic cells with irregular nuclei admixed with small lymphocytes and eosinophilic granulocytes were observed, raising suspicion for a histiocytic disorder. Immunohistochemical stains highlighted expression of CD1a, Langerin (CD207) and S-100 (Fig. 6a, b, c), consistent with the diagnosis of Langerhans cell histiocytosis. Molecular studies showed absence of mutations in Exon 15 of the *BRAF* gene (i.e. no BRAF V600E mutation), instead a 6 base pair deletion in exon 3 of the *MAP2K1* gene (p.E102_I103del) was found. A bone marrow biopsy ruled out infiltration by LCH. Lumbal puncture revealed no evidence of malignant cells.

### Therapy and surgical intervention

**Fig. 7:**
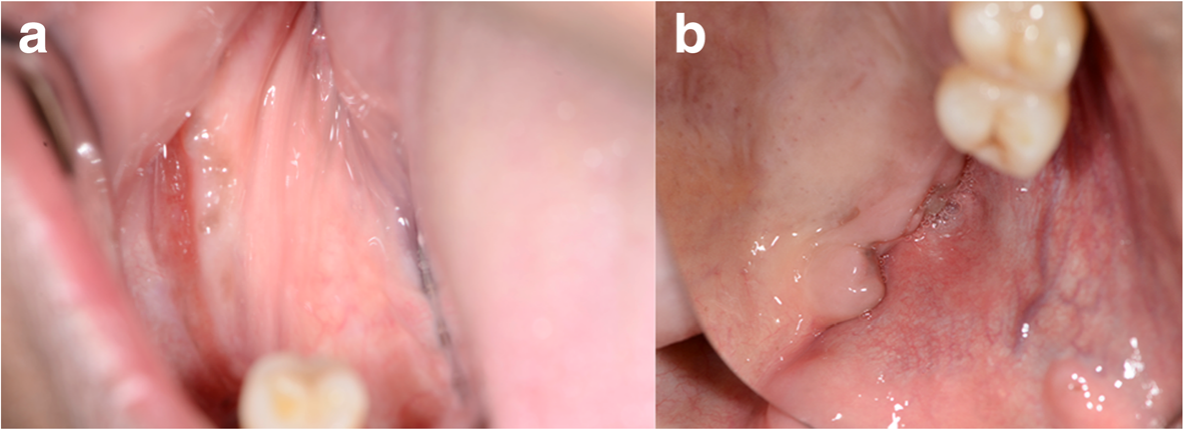
Clinical presentation of right crestal mandibular (**a**) and left crestal maxillary (**b**) gingiva at follow-up after eight months of chemotherapy

**Fig. 8:**
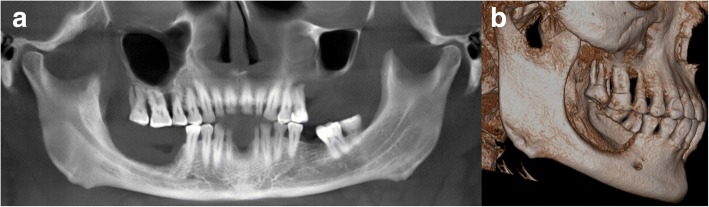
The follow-up CBCT shows bone regeneration in the right mandible and the left maxilla in a reconstructed panoramic view (**a**) and bone regeneration in the right mandible in a 3D-reconstruction (**b**)

**Fig. 9:**
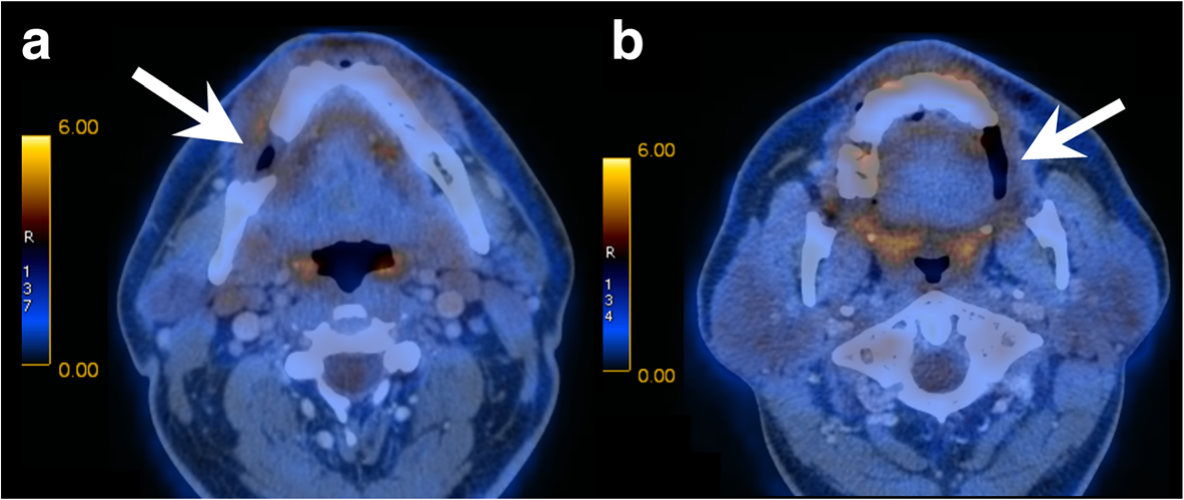
Follow-up FDG-PET/CT shows absence of pathologic radiotracer uptake within the lytic lesions in the right mandible (**a**, arrow) and the left maxilla (**b**, arrow)

Diabetes insipidus centralis, which was persisting since eight years, was defined as having been caused by the LCH. The interdisciplinary tumour board, based on the extension of the disease with affection of the skull base, decided to treat the patient with chemotherapy (Cytarabine). As this disease is rare in adults, there is currently no randomized clinical trial in adults and no therapeutic standard is defined. Retrospective data indicates that Cytarabine is more effectively compatible in adults with LCH with predominant involvement of the bone and the central nervous system compared to the pediatric standard of Vinblastin/Prednison [11]. Prior to this treatment, tooth 48 was extracted due to extensive perifocal bone resorption. The chemotherapy was conducted with Cytarabin (150 mg/m2 day 1−5 intravenous during one hour, repetition day 29). Further a PET/CT was taken two and six months after the start of the chemotherapy. After that, PET/CT’s were taken every 6 months. After six cycles of chemotherapy, the patient clinically showed complete soft tissue healing (Fig. 7a, b) and stable bone lesions in the CBCT (Fig. 8a, b). FDG-PET/CT showed a significant metabolic response of lesions (Fig. 9a, b). Overall, the patient primarily received 12 cycles of chemotherapy, and due to residual activity, another six cycles of chemotherapy. Two years after diagnosis the patient is in good general condition with no signs of recurrent disease.

## Discussion and conclusions

LCH is a rare disease and by virtue of this, at great risk of being under- or misdiagnosed. It is therefore of utmost importance that LCH is kept in mind when dealing with unclear osteolytic bone lesions, especially in the absence of exposure to drugs associated with osteonecrosis (e.g. bisphosphonates). In our case report two biopsies were necessary to diagnose the patient’s condition, highlighting the challenge of sampling error. Bone scan may be falsely negative in osseous LCH. FDG-PET/CT was shown to be a reliable tool in the assessment of patients with various histiocytic diseases, including LCH. FDG-PET/CT can identify active lesions, guide biopsy, and assess the response to treatment [12, 13]. Literature on manifestations in the oral region is limited and mostly consists of case reports or retrospective series [14–16]. Surgical treatment has been shown to be very effective in the treatment of localised oral manifestations of LCH and is usually regarded as being sufficient as a sole treatment, sometimes combined with steroid injections [16, 17]. If surgical removal of the affected area is not possible or if LCH reoccurs in the same location, radiation therapy may serve as an alternative or second line treatment [18]. In severe cases with organ dysfunction or systemic LCH manifestations, chemotherapy significantly improves the outcome, however due to the rareness of the disease, it is still unclear which regimens are best suited for different clinical situations [11]. With the recent discovery of the molecular pathology of the disease, targeted therapy might represent a future treatment option [19]. As other rare diseases, international collaborations are needed to conduct clinical trials, The Histiocyte Society (Histiocyte Society Home. Available at: http://www.histiocytesociety.org. Accessed 25.05.2017) regularly undertakes clinical trials to improve treatment protocols (e.g. LCH-III study) [20]. LCH may be self-limiting or locally recurrent, but high risk and systemic cases can have fatal outcomes, highlighting the importance of optimal management, in accordance to international guidelines.^15^

LCH is a rare disease and optimal management requires interdisciplinary collaboration between specialists (oral surgeon, dentist, ENT, radiologist, pathologist and oncologist). In case of unclear osteolytic changes of the jawbone or unexplained diabetes insipidus centralis, LCH should be considered as a possible cause and biopsy should be sought to establish a definitive diagnosis. Possible differential diagnoses are other cutaneous histiocytosis, bone metastases or malignant tumours and should be considered. Surgical treatment of LCH is effective in localised disease, but radical excision is discouraged. In systemic cases, relapses or high-risk disease systemic chemotherapy is the treatment of choice.

## Abbreviations

CBCT: Cone beam computed tomography
ENT: Ear, nose and throat
FDG: F-fluoro-*D*-deoxyglucose
LCH: Langerhans cell histiocytosis
MRI: Magnetic resonance imaging
PET/CT: Positron emission tomography/computed tomography
